# Gene Variants Involved in Nonsense-Mediated mRNA Decay Suggest a Role in Autism Spectrum Disorder

**DOI:** 10.3390/biomedicines10030665

**Published:** 2022-03-13

**Authors:** Ana Rita Marques, João Xavier Santos, Hugo Martiniano, Joana Vilela, Célia Rasga, Luísa Romão, Astrid Moura Vicente

**Affiliations:** 1 Departamento de Promoção da Saúde e Doenças Não Transmissíveis, Instituto Nacional de Saúde Doutor Ricardo Jorge, Avenida Padre Cruz, 1649-016 Lisboa, Portugal; a.rita.marques@insa.min-saude.pt (A.R.M.); joao.xavier@insa.min-saude.pt (J.X.S.); hugo.martiniano@insa.min-saude.pt (H.M.); joana.vilela@insa.min-saude.pt (J.V.); celia.rasga@insa.min-saude.pt (C.R.); 2 BioISI-Biosystems & Integrative Sciences Institute, Faculty of Sciences, University of Lisboa, Campo Grande, C8, 1749-016 Lisboa, Portugal; luisa.romao@insa.min-saude.pt; 3 Departamento de Genética Humana, Instituto Nacional de Saúde Doutor Ricardo Jorge, Avenida Padre Cruz, 1649-016 Lisboa, Portugal

**Keywords:** autism spectrum disorder, nonsense-mediated mRNA decay, single nucleotide variants, copy number variants

## Abstract

Autism Spectrum Disorder (ASD) is a heterogeneous neurodevelopmental condition with unclear etiology. Many genes have been associated with ASD risk, but the underlying mechanisms are still poorly understood. An important post-transcriptional regulatory mechanism that plays an essential role during neurodevelopment, the Nonsense-Mediated mRNA Decay (NMD) pathway, may contribute to ASD risk. In this study, we gathered a list of 46 NMD factors and regulators and investigated the role of genetic variants in these genes in ASD. By conducting a comprehensive search for Single Nucleotide Variants (SNVs) in NMD genes using Whole Exome Sequencing data from 1828 ASD patients, we identified 270 SNVs predicted to be damaging in 28.7% of the population. We also analyzed Copy Number Variants (CNVs) from two cohorts of ASD patients (N = 3570) and discovered 38 CNVs in 1% of cases. Importantly, we discovered 136 genetic variants (125 SNVs and 11 CNVs) in 258 ASD patients that were located within protein domains required for NMD. These gene variants are classified as damaging using *in silico* prediction tools, and therefore may interfere with proper NMD function in ASD. The discovery of NMD genes as candidates for ASD in large patient genomic datasets provides evidence supporting the involvement of the NMD pathway in ASD pathophysiology.

## 1. Introduction

Autism Spectrum Disorder (ASD) is a clinically heterogeneous neurodevelopmental disorder characterized by impaired social communication skills along with repetitive and restricted behaviors and interests [1]. ASD is relatively common, with a median estimated prevalence worldwide of 1–2% [2], and has a major social and economic impacts in families and society. The pathophysiology of this disease is still unclear, precluding the development of effective therapies.

According to heritability studies, genetic factors account for 50 to 80% of the familial ASD risk [3,4], but genetic determinants are still not fully known. Many studies have shown that Copy Number Variants (CNVs) and Single Nucleotide Variants (SNVs) are associated with an increased risk of developing ASD. *De novo*, inherited, rare and common variants in hundreds of genes have been implicated in the etiology of this disease [5,6,7,8,9,10,11]. Alterations in gene expression [12] and interactions between genetic and environmental risk factors [13] have also been documented in ASD. These factors are quite variable and seem to converge in the phenotypic spectrum of autism, but still do not explain all ASD cases. There is also evidence supporting a clinical and genetic overlap between ASD and other Neurodevelopmental Disorders (NDD) [10,14,15,16], such as Attention Deficit Hyperactivity Disorder (ADHD), Developmental Delay (DD) and Intellectual Disability (ID), and also with Neuropsychiatry Disorders (NPD), including Bipolar Disorder and Schizophrenia [10,17,18]. Considering the high genotypic and phenotypic heterogeneity of ASD, we postulate that important regulatory mechanisms may be modulating genetic expression and clinical presentation. Most ASD candidate genes identified so far are involved in neuronal communication and gene expression regulation, including synaptic proteins, chromatin regulators and transcription factors, and are expressed during brain development [8,10]. Therefore, the discovery of disrupted genes involved in regulatory pathways may be particularly relevant in idiopathic cases.

**Figure 1:**
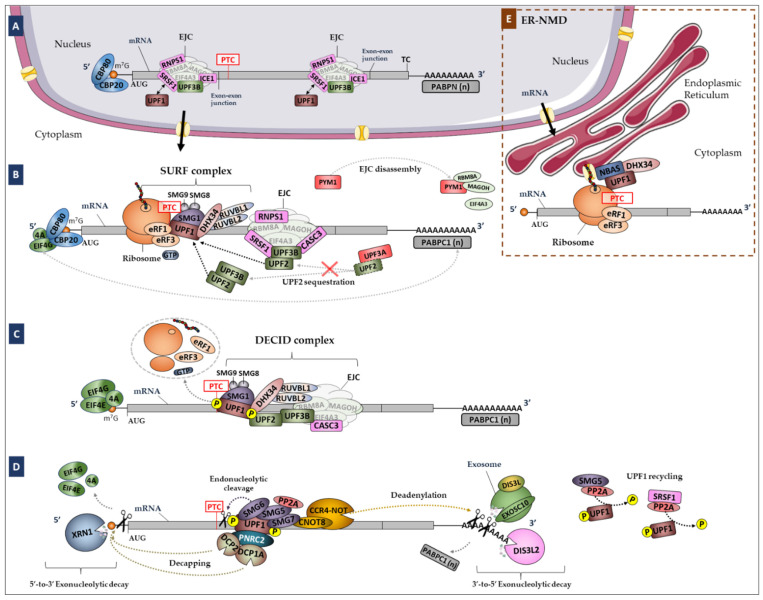
Simplified representation of the NMD pathway in mammalian cells. (**A**) The Exon Junction Complex (EJC; composed by eIF4A3, RBM8A and MAGOH) is formed during splicing and deposited 20–24 nucleotides (nts) upstream of exon–exon junctions, remaining associated with the mRNA during its transport to the cytoplasm. (**B**) In the cytoplasm, the mRNA is translated by the ribosome. If the mRNA carries a premature translation termination codon (PTC), during the pioneer round of translation, i.e., while the mRNA is still bound to the cap binding complex CBP80/20, the ribosome stops at the PTC. If the PTC is located more than 50–54 nts upstream of the last exon–exon junction, the ribosome is not able to displace downstream EJCs. Instead, when the ribosome stops at the PTC, UPF1 can interact with eRF3a, inducing premature translation termination that triggers NMD. For that, the SURF complex is formed by UPF1, eRF1, eRF3a, the SMG1c kinase complex and DHX34 associated with RUVBL1 and RUVBL2. At this stage, both PYM1 and UPF3A can act as NMD repressors. (**C**) UPF2 and UPF3B, either diffused in the cytoplasm (in case of EJC-independent NMD) or bound to the EJC, interact with UPF1, favoring its phosphorylation by SMG1 and the formation of the DECID complex. (**D**) Phosphorylated UPF1 recruits SMG6, which cleaves the mRNA near the PTC, and the SMG5-SMG7 dimer, which recruits the decapping complex DPC2-DCP1a through PNRC2 and the deadenylation complex CCR4-NOT through CNOT8. SRSF1 and SMG5-SMG7 independently recruit PP2A, which dephosphorylates UPF1. NMD-targeted mRNAs are further degraded by 5′-to-3′ and 3′-to-5′ exonucleolytic activities of XRN1 and the exosome, respectively. (**E**) Model of the ER-NMD pathway. The NMD model was adapted from Nogueira et al., 2021 [29]. Legend: NMD, Nonsense-Mediated mRNA Decay; PTC, Premature Termination Codon; EJC, Exon Junction Complex; SURF, SMG1-UPF1-eRF1-eRF3 complex; DECID, Decay Inducing Complex; PP2A, Protein Phosphatase 2A; ER-NMD, NMD response at the Endoplasmic Reticulum; TC, Termination Codon.

Nonsense-Mediated mRNA Decay (NMD) is a conserved genetic RNA quality control mechanism in eukaryotes, responsible for targeting mRNAs harboring premature termination codons (PTCs) for degradation (Figure 1). Thus, the NMD pathway is essential to minimizing the expression of truncated proteins that could be toxic to the cell. NMD also regulates the cellular abundance of normal and fully functional mRNAs, targeting ~10% of the mammalian transcriptome [19]. Consequently, NMD plays a critical role during brain development and neuronal differentiation, and contributes to the regulation of synaptic plasticity and cognitive function [20,21,22,23,24]. Disturbance of this control mechanism can lead to several pathologies, including neurodevelopmental disorders [25].

There are several studies suggesting that NMD may be involved in ASD. For instance, mutations in the *UPF3B* gene, a core NMD factor, were identified in patients that display autistic features [14,15,26,27] and missense mutations in this gene were shown to cause NMD impairment leading to altered neuronal differentiation [20,21]. Recently, Kurosaki and colleagues found that loss of the fragile X mental retardation protein (FMRP), the encoded protein of the *FMR1* gene that represents a leading cause of intellectual disability and autism, results in hyperactivated NMD and gene expression misregulation in early stages of neurogenesis [28].

In this study, we hypothesized that mutations in genes encoding NMD factors and important NMD regulators (Figure 1) may impair the normal function of this mechanism and be involved in the pathophysiology of ASD. To test this hypothesis, we searched for SNVs and CNVs in 46 key NMD genes in a large cohort of patients diagnosed with ASD. Our results support a role of NMD in ASD, due to the identification of genetic variants located on functional regions of NMD genes in 258 ASD cases. This work reveals novel ASD candidate genes, such as *UPF3A*, *SMG1*, *SMG5*, *SMG7*, *NBAS*, *DHX34* and *ICE1*, which have important functions in the NMD pathway.

## 2. Materials and Methods

**Figure 2:**
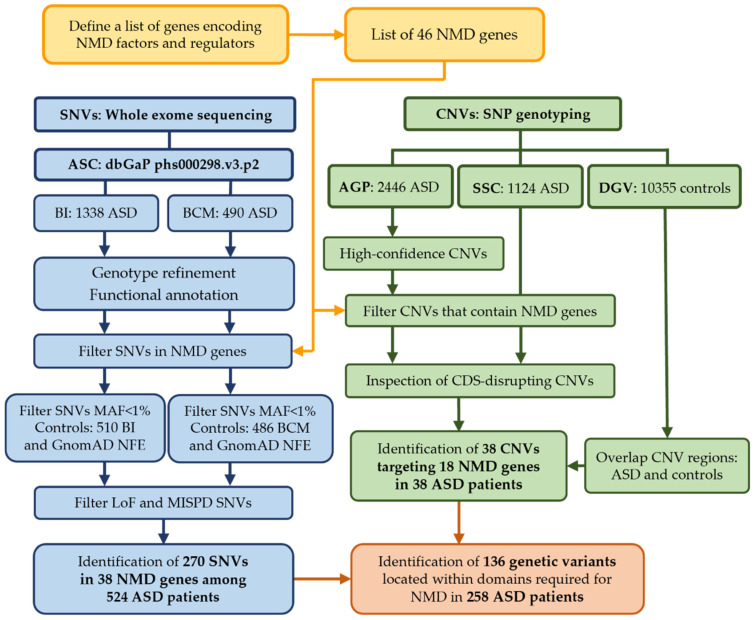
Overview of the analysis. The study began with the identification of 46 genes (Table 1) encoding proteins involved in the NMD pathway or in its regulation (yellow) that were used for further analyses. This workflow describes the analysis of SNVs obtained from ASC WES datasets for 1828 ASD patients (1338 BI and 490 BCM, blue) and the analysis of CNVs predicted from SNP genotyping data in 3570 ASD patients from AGP (2446) and SSC (1124) datasets (green). Analysis proceeded separately for CNVs and SNVs to identify variants in NMD genes and ended up with the identification of 270 SNVs (Figure 3) and 38 CNVs (Table 2) in 524 and 38 ASD probands, respectively. Protein domains affected by SNVs or CNVs were then identified (orange), and a total of 136 genetic variants were located within regions required for NMD in 258 ASD patients (Table 3). Legend: SNVs, Single Nucleotide Variants; CNVs, Copy Number Variants; ASC, Autism Sequencing Consortium; AGP, Autism Genome Project; SSC, Simons Simplex Collection; BI, Broad Institute; BCM, Baylor College of Medicine; DGV, Database of Genomic Variant; CDS, coding sequence for protein; MAF, Minor Allele Frequency; NFE, Non-Finnish European; MISPD, missense variants predicted to be damaging and deleterious; LoF, loss-of-function.

The overall methodology is represented in Figure 2 and is described in detail below.

### 2.1. Identification of Genes Encoding NMD Factors and Regulators

To compile a list of human genes encoding proteins involved in the NMD mechanism, we searched recent reviews in PubMed, using the term “nonsense mediated decay” and article type “Review”. Then we enriched this gene list using the web-based open-access resources AmiGO (http://amigo.geneontology.org/amigo [30]) and Reactome (https://reactome.org/). Gene Ontology (GO) enrichment analysis was performed by querying the AmiGO database with the term “nonsense mediated decay”, and the output files containing “gene and gene products” results, identified in *Homo sapiens*, were downloaded on 9 September 2020. Reactome was also interrogated using the term “nonsense mediated decay” to retrieve the genes involved in NMD pathway (ID: R-HSA-927802) on 28 September 2020. We identified 122 genes through AmiGO, 116 genes through Reactome (Supplementary Tables S1 and S2) and 33 genes through a literature review, some of which were redundant, as expected. After manually curating this gene list, we ended up with a list of 46 experimentally validated genes that encode proteins involved in NMD (hereafter called NMD genes). Genes were divided into groups according to their functions in NMD: EJC—Exon Junction Complex (EJC) factors and regulators; SURF-DECID—components of SMG1-UPF1-eRF1-eRF3 (SURF) and decay inducing (DECID) complexes; mRNA decay—NMD mRNA decay phase; ER-NMD—NMD response at the Endoplasmic Reticulum (ER); Regulator—involved in NMD regulation. Gene symbols and names are in accordance with HUGO Gene Nomenclature Committee (HGNC) guidelines [31].

### 2.2. ASD Genomic Datasets

To identify genetic variants within NMD genes in ASD patients, we analyzed SNV and CNV data from ASD and control populations published in previous studies.

The SNV dataset was obtained from the Autism Sequencing Consortium (ASC) [32] deposited in dbGaP (ARRA, Autism Sequencing Collaboration; dbGaP Study Accession: phs000298.v3.p2). This dataset contains Whole Exome Sequencing (WES) data from two sources: 490 ASD cases and 486 unrelated controls sequenced using the Solid platform and called with AtlasSNP 2 at Baylor College of Medicine (BCM) [33], and 1338 ASD cases and 510 unrelated controls sequenced using the Illumina platform and called with GATK at Broad Institute (BI) [8]. In total, we analyzed WES for 1828 ASD patients (1451 males and 356 females).

The CNV datasets of ASD patients were obtained from two different studies: 2446 patients (2114 males and 322 females) from The Autism Genome Project (AGP) [6,34] and 1224 patients (967 males and 157 females) from the Simons Simplex Collection (SSC) [35]. We analyzed the CNV calls from the AGP Consortium deposited in dbGaP (Stage I and II, dbGaP Study Accession: phs000267.v5.p2) and a dataset of rare and high-confidence CNVs that passed quality control from the study by Sanders et al., 2011 [5]. Two CNV datasets from unrelated controls (Cooper 2011 and Shaikh 2009) were inspected for variant frequencies (10,355 individuals with no history of neuropsychiatric disease) in the Database of Genomic Variant (DGV: http://dgv.tcag.ca/dgv/app/home, last updated on 15 May 2016). ASD patients from the ASC, AGP and SSC met the criteria for autism or ASD using the Autism Diagnostic Observation Schedule (ADOS) and Autism Diagnostic Interview-Revised (ADI-R) [6,32,34,35].

### 2.3. Sequencing Data Processing, Annotation and SNV Discovery

To exclude artifacts often observed due to sequencing errors, we applied quality filtering and genotype refinement to the raw VCFs using Genome Analysis Toolkit (GATK, version 3.7) for BCM and BI separately [36]. Multi-allelic sites were split into bi-allelic sites, and we only considered variants with a minimum depth filter (DP > 8) and genotype quality (GQ > 20) for all events. We only considered SNVs in ASD cases that were rare in controls from ASC (MAF < 1%). After evaluation of data quality, variants were functionally annotated with Variant Effect Predictor (VEP, version 86) [37] using the human genome build 37 (GRCh37/hg19) as reference. VEP assigned properties to the variants, including gene name; consequence type; pathogenicity predictions (Polyphen, SIFT, Combined Annotation-Dependent Depletion (CADD) [38]; probability of being loss-of-function intolerant (pLI) [39] and missense Z (mis_Z) [40] scores); and the allele frequencies observed in controls from the Genome Aggregation Database (gnomAD, v2.2.1). For this work we focused on SNVs in NMD genes identified in ASD patients. Variants with predicted damaging effects were classified as loss-of-function (LoF—variants which include frameshifts, stop gains, stop losses and splicing ≤ 2 bp) or probably damaging and deleterious missense (MISPD) variants (defined by Polyphen [41] and SIFT [42]). We further investigated whether these variants were present in controls from the sub-population of Non-Finnish European (NFE) in gnomAD, and further analyzed only the variants that were rare (MAF < 1%) or not detected in gnomAD.

### 2.4. CNV Discovery

CNV discovery was previously performed using Illumina SNP genotyping data as described in the original papers [5,6,34]. Only rare CNVs (<1% frequency) called by at least two of three algorithms were considered in this analysis. First, we identified CNVs encompassing NMD genes. Then we manually inspected these CNVs to characterize the extension of the deletions or amplifications encompassing the coding sequence (CDS) of each NMD gene. Finally, we assessed whether these CNVs were also observed in DGV controls.

### 2.5. Protein Domains

There are known conserved protein domains that display important functions in NMD. To assess whether the variants predicted to be damaging in this study impact important protein domains of NMD genes, SNVs and CNVs were manually curated, and protein domains were identified. We used annotation with the Universal Protein Resource (UniProt) database and reviewed literature to identify protein domains.

### 2.6. ASD Candidate Genes

To examine if NMD genes were previously reported as candidate genes for ASD, we compared our list of NMD genes with a list of ASD susceptibility genes. A list of 1003 ASD candidate genes was downloaded from the manually curated SFARI Gene database (https://gene.sfari.org/ accessed on 8 March 2021), released on 13 January 2021. This comprehensive database contains up-to-date information on genes linked to ASD risk, and based on the evidence supporting association with ASD, genes are ranked into four categories: syndromic (126 genes), high confidence (207 genes, category 1), strong candidates (211 genes, category 2) and suggestive evidence (506 genes, category 3). Additionally, to identify ASD candidates that are known NMD targets, we compared SFARI gene list with a list of high-confidence neuronal NMD targets obtained from Kurosaki et al., 2021 [28].

### 2.7. Brain Expression of NMD Genes

We used gene expression data to assess whether NMD genes were expressed in adult human brain and during the early stages of human brain development. For that we obtained gene expression data from two open-source resources: The Human Protein Atlas [43] and the Expression Atlas from European Bioinformatics Institute (EMBL-EBI). The Brain Atlas subset within Human Protein Atlas was downloaded and proteins expressed in Human Adult brain were identified (https://www.proteinatlas.org/, last updated on 24 February 2021). We also used expression data from The Human Developmental Biology Resource (HDBR) [44] which contains baseline gene expression from different brain regions across a substantial period of early development (4 to 20 post-conception weeks [PCW]) and was deposited in the EMBL-EBI Expression Atlas (E-MTAB-4840, 15 May 2019). EMBL-EBI makes available the normalized counts per gene (TPMs) and defines the following cut-offs: no expression (below cutoff; TMP < 0.5), low expression (between 0.5 to 10 TPM), medium expression (between 11 to 1000 TPM) and high expression (above 1000 TPM). We used these criteria to classify gene expression of our candidate genes.

## 3. Results

### 3.1. Genes Encoding Proteins Involved in the NMD Pathway

We identified 46 experimentally validated genes encoding core NMD factors and regulators (Table 1 and Figure 1) through manual curation of a gene list obtained by enrichment analysis and literature review.

All 46 NMD genes are expressed in the human adult brain, as confirmed by the Human Protein Atlas. We also examined the EMBL-EBI Expression Atlas to assess whether these genes are expressed during early brain development, and we confirmed that all genes are expressed in the forebrain, midbrain and hindbrain in the first 4 to 8 post-conception weeks (PCW) and in the cerebral cortex in the age range of 8–17 PCW. Additionally, we observed that the expression levels of these genes vary during different stages of neurodevelopment. For example, the *UPF1*, *DHX34* and *RUVBL2* genes presented lower baseline expression at 8 PCW, whereas *SRSF1* presented higher baseline expression, when compared to the overall transcriptome.

#### Evidence for NMD Involvement in ASD Pathophysiology

From the list of 46 NMD genes, we found that seven are previously known ASD candidate genes described in the SFARI gene database, classified as syndromic (*PPP2CA*), high confidence (*UPF3B* and *FMR1*) or suggestive evidence (*EIF4E*, *EIF4G1*, *SMG6* and *UPF2*). Additionally, we overlapped the list of 1003 ASD candidate genes from SFARI with a list of 1277 high-confidence neuronal NMD targets from a previously published study [28] to understand whether the expression of known ASD risk genes might be regulated by NMD. We found that 71 ASD risk genes (Supplementary Table S5) are upregulated upon UPF1 knockdown and bound to hyperphosphorylated UPF1 in neuronal cells (see Kurosaki et al., 2021 [28]). Overall, this analysis indicated that NMD might be important to regulating the expression of ASD risk genes in the brain.

### 3.2. Discovery of SNVs in NMD Genes

**Figure 3:**
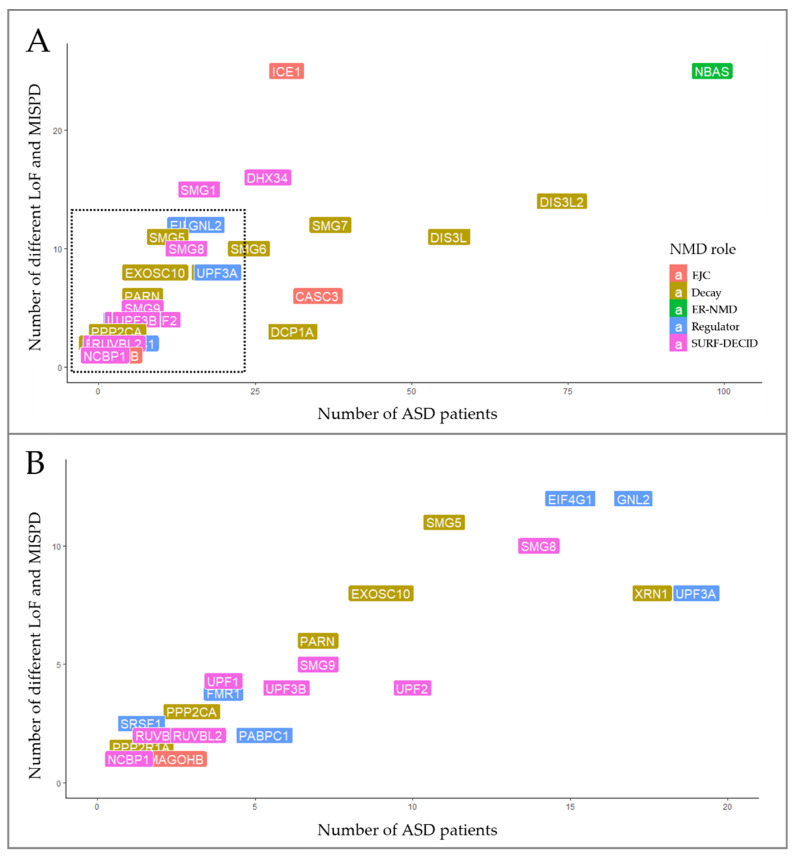
Distribution of rare SNVs predicted to be damaging among 38 NMD genes in ASD patients. (**A**) Variants in NMD genes were identified in 1 to 5% of ASD patients. (**B**) Detailed distribution of gene variants in <1% of the ASD population sample.

We explored WES data from 1828 ASD patients and retrieved a total of 4922 SNVs in the 46 NMD genes. Following variant prioritization, we identified a total of 270 SNVs predicted to be damaging located on 38 NMD genes, in 28.7% (524/1828) of the ASD patients (Supplementary Table S3). These variants were almost equally distributed among males (416/1451, 28.7%) and females (99/356, 27.8%). The number of unique SNVs identified in ASD patients among the 38 NMD genes are represented in Figure 3. These variants were either rare (MAF < 1%) or not observed in gnomAD controls, and 11.5% (31/270) were classified as LoF and 88.5% (239/270) as MISPD. Among LoF variants, we discovered 6 frameshift, 10 nonsense and 15 splicing variants in 20 NMD genes (Supplementary Table S3).

**Figure 4:**
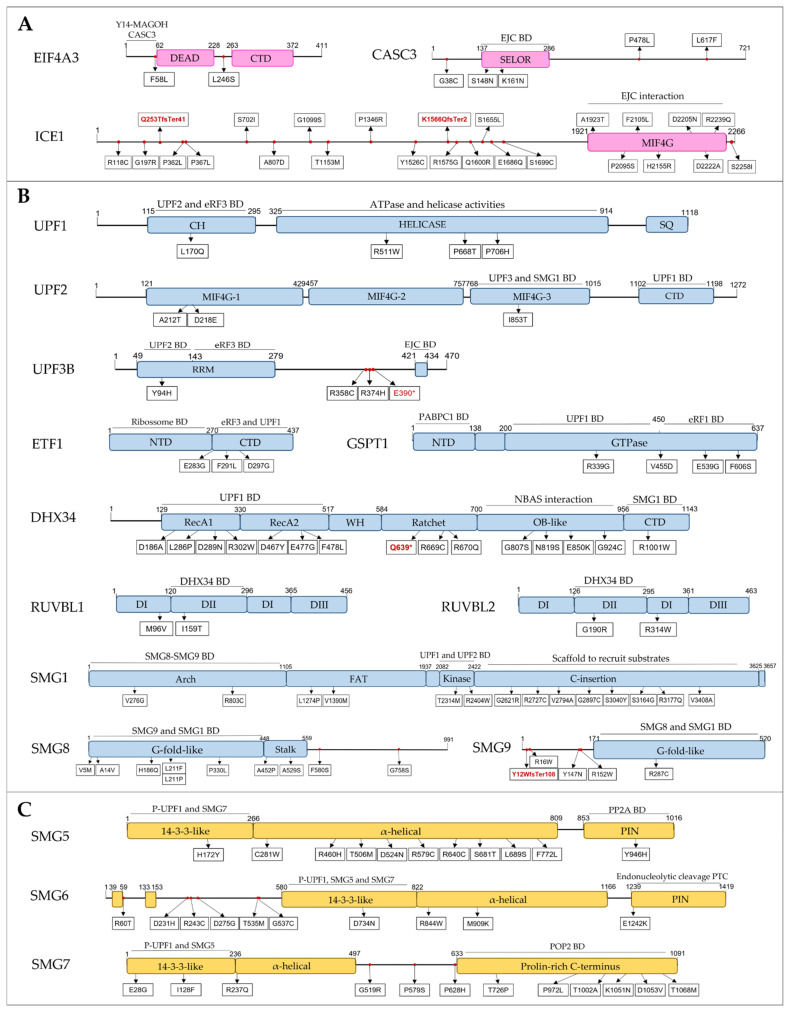

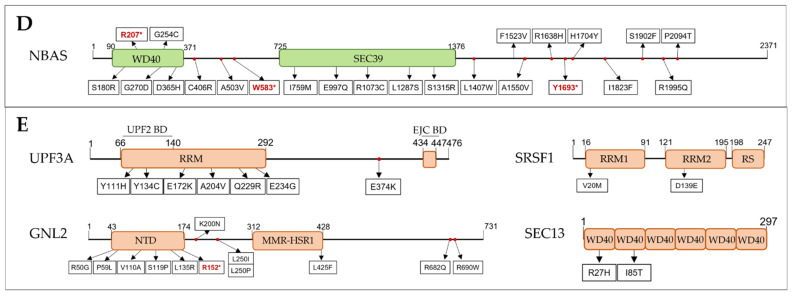
Schematic domain architecture of proteins involved in NMD, and representations of the protein alterations encoded by MISPD and nonsense/frameshift variants identified in ASD subjects. The binding regions for interacting proteins are indicated. Proteins were grouped by NMD groups: (**A**) EJC, (**B**) SURF-DECID, (**C**) mRNA decay, (**D**) ER-NMD and (**E**) Regulator. Legend: MISPD, missense variants predicted to be damaging and deleterious; BD, Binding Domain; CH, cysteine and histidine-rich domain; SQ, serine- and glutamine-rich domain; RS, arginine and serine-rich domain; RRM, RNA recognition motif; EJC, Exon Junction Complex; PIN, PilT N-terminus; P-UPF1, phosphorylated UPF1; WH, Winged-Helix; CTD, C-terminal domain; NTD, N-terminal domain.

Interestingly, the *NBAS* gene stood out for having twenty-five different variants (20 MISPD, 3 nonsense and 2 splicing) identified in 5.4% (98/1828) of the ASD patients (Figure 3). From these, 10 SNVs were located on two predicted functional domains (the secretory pathway protein Sec39 and N-terminal WD40 repeats domains), 10 SNVs were in the C-terminal region, one variant was predicted to affect splicing within N-terminal region and 3 SNVs were located upstream of the Sec39 domain (Figure 4D). The *NBAS* gene is not constrained (pLI = 0, mis_Z < 0) but is important for the NMD response at the ER [78]; however, the required domains for NMD are still not known. Moreover, we identified 97 unique SNVs in 8 genes that were present in 1 to 4% of the ASD population: *DIS3L2* (4%, 74/1828), *DIS3L* (3%, 56/1828), *SMG7* (2%, 37/1828), *CASC3* (1.9%, 35/1828), *DCP1A* (1.7%, 31/1828), *ICE1* (1.6%, 30/1828), *DHX34* (1.5%, 27/1828) and *SMG6* (1.3%, 25/1828) (Figure 3, Supplementary Table S3).

To evaluate the impact of SNVs in NMD genes identified in ASD cases, we performed a grouped analysis of overall LoF and MISPD variants. Among the five groups of NMD genes, we found that 34.4% (93/270) of the SNVs mapped to thirteen mRNA decay genes, 26.3% (71/270) mapped to twelve SURF-DECID genes, 17% (46/270) mapped to eight regulator genes, 13% (35/270) mapped to four EJC genes and 9.3% (25/270) mapped to one ER-NMD gene (Figure 3 and Supplementary Table S3).

Through the analysis of the protein domains affected by these variants, we identified 125 SNVs within gene regions encoding protein domains known to be important for NMD function, in 13.5% (247/1828) of the ASD patients. Most of these variants were identified in constrained genes with pLI ≤ 0.5, and some of these genes are extremely intolerant to LoF (pLI > 0.9) and missense variants (mis_Z > 3.09) [39,40]. For some genes, namely, *DCP1A*, *DCP2*, *DIS3L*, *DIS3L2*, *EXOSC10*, *MOV10*, *PARN*, *PPP2R1A*, *PPP2CA* and *XRN1*, we were not able to identify the SNVs that could influence NMD function because the protein domains necessary for NMD are not described.

#### 3.2.1. EJC Components and Regulators

We discovered 34 SNVs (2 splicing, 2 frameshift and 30 MISPD) in three genes encoding EJC components, *EIF4A3*, *CASC3* and *ICE1*, in 68 ASD patients (Supplementary Table S3). Three MISPD are located within the N-terminal and the SELOR domain of *CASC3* gene, seven MISPD are located on the functional MIF4G domain of the *ICE1* gene, and two frameshift variants are predicted to cause premature termination of translation upstream the MIF4G domain of the *ICE1* gene (Figure 4A). CASC3 (also known as BTZ) promotes the degradation of NMD substrates by interacting with EJC via its SELOR domain [49], a mechanism that also requires the N-terminus domain [50]. The C-terminal MIF4G domain of ICE1 interacts with eIF4A3 to promote UPF3B-EJC association [51]. Additionally, one MISPD was located in the *EIF4A3* N-terminal domain, which is required to trigger NMD [45]. Importantly, *ICE1*, *CASC3* and *EIF4A3* are constrained genes (pLI ≥ 0.5).

#### 3.2.2. SURF and DECID Complexes

We discovered a total of 12 variants in *UPF1*, *UPF2* and *UPF3B* genes in 20 ASD patients (Supplementary Table S3). From these, we found 1 splicing and 8 MISPD variants located on functional protein domains: 3 within the helicase domain and 1 in the regulatory CH rich domain of UPF1, 4 within the MIF4G domains of UPF2 and 1 located on RNA recognition motif (RRM), necessary for UPF2-binding, of UPF3B (Figure 4B). Additionally, we discovered three variants in a region between RRM and EJC-binding domains of the *UPF3B* gene. The helicase and CH rich domains of UPF1 protein are required for ATPase and helicase activities [87] and for UPF2-binding [46], respectively. The MIF4G-1 and MIF4G-2 domains of UPF2 have a key scaffolding role, and the MIF4G-3 domain is required for UPF3 and SMG1-binding, thereby being the essential module for triggering NMD [88].

Regarding the SMG1c kinase complex, we found 15 SNVs (14 MISPD and 1 splicing) located on four functional *SMG1* domains: Arch, FAT, Kinase and C-Insertion domains; 8 SNVs located within two functional *SMG8* domains: G-fold-like and Stalk domains; and two SNVs (1 frameshift and 1 MISPD) predicted to affect the G-fold-like domain of *SMG9* (Figure 4B). From these, we identified 23 ASD patients with 19 SNVs located in domains required to form the SMG1-SMG8-SMG9 complex and to activate NMD through UPF1 phosphorylation: Arch, SMG1 kinase and C-Insertion domains of SMG1 protein and G-fold domains of SMG8 and SMG9 proteins [61]. Among SURF components, we discovered 1 splicing and 3 MISPD variants located on C-terminal domain of the *ETF1* gene, necessary for eRF3a and UPF1 binding, and 4 MISPD variants within the GTPase domain of *GSPT1* gene, required for eRF1 and UPF1 binding (Figure 4B). Moreover, we identified 1 splicing, 1 nonsense and 14 MISPD variants located within four functional protein domains of *DHX34* gene: helicase, ratchet, OB-like and C-terminal domains; and two MISPD variants in *RUVBL1* and *RUVBL2* genes located on DII domains (Figure 4B). DHX34 binds UPF1 and SMG1 through its helicase and C-terminal domains, respectively, promoting NMD [64], and its OB-like domain is required for its interaction with NBAS [78]. The RUVBL1-2 DII domains are important for DHX34 binding [66], and thus are involved in NMD. Importantly, *UPF1*, *UPF2*, *UPF3B*, *SMG1*, *ETF1*, *GSPT1*, *RUVBL1* and *RUVBL2* are constrained genes (pLI ≥ 0.5).

#### 3.2.3. mRNA Decay

Thirty-three variants were discovered in *SMG5, SMG6* and *SMG7* genes in 60 ASD patients (Supplementary Table S3). Of these, 12 MISPD variants were located in functional protein domains essential for NMD: 2 variants in the 14-3-3-like and PIN domains of SMG5, 2 variants in the 14-3-3-like and PIN domains of SMG6 and 8 variants in the 14-3-3-like and proline-rich C-terminus domains of SMG7 (Figure 4C). The 14-3-3-like domain of SMG6 and the SMG5–SMG7 complex associates with phosphorylated UPF1 [54,89], the PIN domain of SMG6 promotes endonucleolytic cleavage near the PTC of a nonsense mRNA [71] and the SMG7 prolin-rich C-terminus interacts with CNOT8 to recruit the CCR4–NOT complex. *SMG6* and *SMG7* are constrained genes (pLI ≥ 0.5). Additionally, we found 47 variants in genes encoding exoribonucleases involved in NMD [74] in 164 ASD patients. Among these, 35 MISPD variants were located in functional protein domains: CDS1, CDS2 and RNB domains (*DIS3L* and *DIS3L2*); PMC2NT, DNA_pol_A_exo1 and HRDC domains (*EXOSC10*); N-terminus, XRN_D2_D3 domains and Xnr1 SH3-like domain (*XRN1*); and CAF1 domain (*PARN*). We also found eight LoF variants in these genes: 3 nonsense (*DIS3L2*, *EXOSC10* and *PARN*), 3 frameshift (*EXOSC10*, *DIS3L2* and *XRN1*) and 2 splicing (*PARN*, *EXOSC10*). Moreover, we identified 13 SNVs within *MOV10*, *PPP2R1A*, *PPP2CA*, DCP1A and DCP2 genes in 41 ASD patients, of which two were located on HEAT and PP2A subunit C binding domains (*PPP2R1A*) and three were located on CH and helicase domains (*MOV10*). Although these genes are involved in the degradation of NMD targets [74], the required domains to exert their function in NMD are not defined.

#### 3.2.4. NMD Regulators

This group includes the genes encoding proteins known to regulate NMD, but either they do not belong to the core machinery, or their exact functions are still unknown. We discovered eight SNVs within *UPF3A* gene in 19 ASD patients: 1 splicing variant upstream of the EJC-binding domain, 1 MISPD within a region of unknown function and 6 MISPD variants located on the RRM domain, including the region needed for UPF2 binding (Figure 4E). Additionally, one variant was identified in the RRM2 domain of *SRSF1*, which was described to be important for UPF1 dephosphorylation through interactions with SMG7 and PP2A [85], and nine variants (1 nonsense and 8 MISPD) were discovered within functional domains of *GNL2* and *SEC13* (Figure 4E).

### 3.3. CNVs Encompassing NMD Genes in ASD Patients

To further assess the contribution of large deletions and duplications of the 46 NMD genes to ASD, we analyzed CNVs from 3570 ASD patients. We found 38 CNVs encompassing 18 NMD genes in 1% (38/3570) of the ASD patients (34 males and 4 females), of which 8 were CNV losses and 30 CNV gains (Table 2).

We further characterized the extent of the deletion or duplication observed for each gene and analyzed whether the same variant was observed in 10,355 DGV controls (Supplementary Table S4). The majority of CNVs disrupting whole genes or important functional domains were exclusive in cases or observed in only one or two controls. Importantly, we discovered two ASD patients carrying CNVs (partial duplications) that included two regions required for NMD: the PIN domain of SMG6 protein and the MIF4G domains of UPF2 protein [71,88]. Moreover, we identified 10 patients with CNVs encompassing complete genes (*RBM8A*, *UPF3B*, *UPF3A*, *GSPT1*, *NCBP2* and *DHX34*) that may lead to dosage imbalances and thus influence NMD. Both *SMG6* and *UPF2* are highly constrained genes (pLI ≥ 0.9); and *RBM8A*, *UPF3B* and *GSPT1* genes are also constrained (pLI ≥ 0.5). The numbers of CNVs and SNVs disrupting genes or gene regions involved in NMD function are shown in Table 3.

## 4. Discussion

Despite enormous efforts in ASD research since its first description, the pathophysiology of this disorder is still unclear. In this study, we tested the hypothesis that genes encoding proteins involved in NMD (Figure 1), a regulatory mechanism that ensures the degradation of PTC-containing transcripts and that controls the expression of some naturally occurring transcripts [19], play a role in ASD.

NMD depends on multiple genes, and through literature review and enrichment analysis we defined a group of 46 experimentally validated NMD factors and regulators, of which seven were previously known as candidate risk genes for ASD. Some of the proteins encoded by these 46 genes, such as *UPF1*, are essential for the overall NMD response, whereas others participate in alternative NMD branches and are involved in the regulation of different mRNA targets. We confirmed that these 46 genes are expressed in the adult human brain and during early neurodevelopment, when the early stages of cortex development occur, and major brain regions are established [90]. This suggests that NMD may be modulated during neurodevelopment, a period of relevance for ASD. Moreover, we identified 71 neuronal ASD risk genes that are experimentally validated NMD targets [28], suggesting that NMD may regulate the expression of ASD risk genes during neurodevelopment. These observations are consistent with previous findings showing that selective mRNA decay is critical for specifying the developmental fate of human embryonic cell lineages [22].

Our study aimed at identifying genetic variants in these 46 genes that might influence the NMD response, including SNVs predicted *in silico* to be damaging and CNVs that might influence gene expression or disrupt gene structure, leading to novel transcripts [91], comparable to what was observed by Nguyen et al., 2013 [92]. Our search for SNVs was carried out in publicly available large ASD datasets with exome sequences, so that we could explore gene variants directly affecting known functional domains involved in the NMD response, namely the variants located in exonic regions or affecting splicing. We additionally inspected CNVs targeting NMD genes in the well characterized AGP and SSC datasets. The comprehensive search for rare SNVs and CNVs in 46 NMD genes among ASD patients led to the discovery of 270 SNVs predicted to be damaging within 38 genes in 28.7% of the ARRA WES population and 38 CNVs located on 18 genes in 1% of the AGP and SSC population. Both SNVs and CNVs were either rare or absent from control datasets from gnomAD or DGV, respectively. In particular, CNVs targeting NMD genes were rare in ASD patients but also extremely rare in controls (MAF < 0.1%).

Analysis of the protein domains affected by SNVs and CNVs revealed that 136 of these genetic variants, identified in 258 ASD patients, may have an impact on NMD function. Most of these variants mapped to constrained genes, indicative of intolerance to LoF and/or missense variation, therefore supporting a damaging impact in gene function. Some of the variants identified in these genes were located on domains previously known to influence NMD activity and/or implicated in ASD. For instance, we discovered SNVs within a region of *UPF3B* gene, located between RRM and EJC-binding domains (Figure 4), where mutations are known to impair NMD function [21]. Baird et al., (2018) previously reported that *ICE1* depletion leads to an increased abundance of NMD targets, including the ASD candidate gene *ANXA1* [8,51,93]. Our findings thus suggest that the variants predicted to be damaging may influence *ICE1* function in NMD, which in turn would affect the expression of NMD targets in ASD patients. The *UPF3A* gene is known to play an important role during development, and inactivation of the *UPF3A* gene results in hyperactivated NMD in mice [83]. The genetic variants identified in the *UPF3A* gene that were predicted to be damaging may influence UPF3A function and NMD activity during neurodevelopment, leading to altered target gene expression. Like *UPF3B*, mutations in *UPF3A* may contribute to ASD pathophysiology, because they exhibit antagonistic effects upon the same mechanism [83]. The *RBM8A* gene (also known as *Y14*) has an equally important role in interneuron development [24]. CNVs targeting the 1q21.1 region where the *RBM8A* gene is located have previously been associated with neurodevelopmental disorders, including ASD [92,94].

Taken together, our research identified gene variants that may interfere with proper NMD function in 13.3% (SNVs) and 0.4% (CNVs) of the ASD patients analyzed in this study. Although these variants are individually rare in ASD cases, overall, they were identified in over 13% of the patients and are located on regions encoding protein domains required for NMD. Some of the NMD genes with relevant variants identified in this study, were previously implicated in ASD or target ASD candidate genes, reinforcing the notion that NMD dysfunction may contribute to the disease. In this work we focused on exonic SNVs with putative direct impact on the protein domains required for efficient NMD. While regulatory regions in NMD genes are not yet fully characterized, they can also contain genetic variants with an impact on NMD function and in the future need to be evaluated in ASD datasets with whole genome sequences. Future studies to experimentally validate the functional consequences of the identified variants on protein function are now mandatory. Additionally, future work should address the expression levels of NMD targets both in neurons and other cell types, to evaluate whether these predicted pathogenic variants influence NMD and to assess if this effect is specific to neuronal cells.

ASD is characterized by a wide spectrum in clinical presentation. Given the large number of targets for the NMD genes we assessed here, several of which are ASD candidates, the variants identified may influence ASD phenotypic heterogeneity. In this study, we did not explore the association of phenotypic presentation within the autism spectrum with the NMD gene variants. This was not possible because homogeneous clinical information was not available in a sufficient number of subjects from these datasets to draw firm conclusions, highlighting the pressing need for making phenotypic data available and interoperable for large disease datasets. There is also a growing body of evidence implicating genes and molecular pathways associated with ASD in other NDD, and even extending common genetic factors to several NPD [10,16,17,18]. Both the clinical and genetic boundaries between ASD and other NDD have many overlaps, with some risk genes being more predominant for ASD and others conferring risk for both ASD and other NDD [10,14,15,16,92,94]. It is therefore not surprising that some of the NMD genes identified in this study have previously been associated with other disorders. For instance, loss-of-function mutations in *UPF2*, *UPF3B*, *SMG8* and *SMG9* genes, and CNVs targeting *UPF2*, *UPF3A*, *SMG6*, *EIF4A3*, *RNPS1* and *RBM8A* genes, have been implicated in a variety of neurodevelopmental disorders, including DD, ID, ADHD and TAR syndrome [14,15,27,92,94,95,96]. NMD is a regulator of many biological processes from the early stages of development to adulthood and occurs in different tissues and cell types, and variants that impair NMD function may contribute to many pathophysiological mechanisms. Future work will need large datasets with extensive genomic and phenotypic information to ascertain the convergence among brain diseases, and determine the contributions of the NMD pathway, as a critical regulator of synaptic plasticity, neural development and neural stem cell differentiation decisions, to neurodevelopmental disorders.

## 5. Conclusions

An efficient NMD function is extremely important to regulate the expression of NMD targets and is essential during neurodevelopment and neuronal differentiation, and for brain function throughout life. Overall, our study provides novel evidence for a contribution of the NMD pathway to ASD. This work has identified, in a significant number of patients, genetic variants that can interfere with NMD function and may contribute to ASD through the misregulation of NMD target gene expression, particularly during neurodevelopment. Moreover, the branched nature of NMD suggests that diverse genetic alterations may have impacts on different NMD pathways and influence the expression of multiple NMD targets, contributing to the phenotypic heterogeneity of ASD. Further studies are needed to experimentally validate the functional impact of the variants found in this study, so that we can better understand the role of the NMD pathways in ASD. A full appreciation of these regulatory mechanisms in ASD and other NDD will constitute an opportunity for the development of much needed therapeutic interventions for these conditions.

## Figures and Tables

**Table 1:** List of human genes encoding NMD factors and regulators.

NMD Group	Gene SYMBOL ^(1)^	Alternative Name ^(2)^	Role in NMD	References
EJC	*EIF4A3*	eIF4AIII DDX48	RNA helicase eukaryotic initiation factor 4A3 is a core EJC factor that interacts with the Y14-MAGOH heterodimer to provide a stable and direct binding site for the UPF3B protein and activate NMD	[45,46]
	*RBM8A*	Y14	RNA-binding motif protein 8A is a core EJC factor that interacts with eIF4A3 and MAGOH to provide a stable and direct binding site for the UPF3B protein and activate NMD	[45,46]
	*MAGOH*	MAGOH1	Mago nashi homolog protein is a core EJC factor that interacts with eIF4A3 and Y14 to provide a stable and direct binding site for the UPF3B protein and activate NMD	[45,46,47]
	*MAGOHB*	MGN2	Mago nashi protein homolog B is a paralog of MAGOH that interacts with eIF4A3 and Y14 forming the trimeric EJC core to activate NMD	[47]
	*RNPS1*		RNA-binding protein S1 is a component of the SR-rich EJCs that enhances NMD in early phase of the pathway	[45,48]
	*CASC3*	MLN51 BTZ	The peripheral EJC component CASC3 activates NMD and promotes SMG6-dependent endonucleolytic cleavage	[48,49,50]
	*ICE1*	KIAA0947	Component of the little elongation complex promotes the association of EJC with UPF3B and activates NMD	[51]
	*PYM1*	WIGB	Ribosome-associated protein PYM interacts with Y14-MAGOH triggering EJC disassembly which leads to NMD inhibition	[52]
SURF-DECID	*UPF1*	RENT1 smg-2	Up-frameshift protein 1 is the central component of the NMD pathway; its helicase and ATPase activities are essential to trigger NMD	[46,53,54]
	*UPF2*	RENT2 smg-3	Up-frameshift protein 2 and UPF3B interact with UPF1 favoring its phosphorylation by SMG1 and formation of DECID complex	[46,53]
	*UPF3B*	UPF3X	Up-frameshift protein 3B and UPF2 interact with UPF1 favoring its phosphorylation by SMG1 and formation of DECID complex; UPF3B also forms a stable trimeric complex with eRF1-eRF3a to promote dissociation of the termination complexes and triggers NMD	[46,53,54,55]
	*ETF1*	eRF1	Eukaryotic release factor 1 is part of the eRF1–eRF3 translation termination complex that associates with UPF1 and SMG1-SMG8-SMG9 to form SURF and activate NMD	[53]
	*GSPT1*	eRF3a	Eukaryotic release factor 3 is part of the eRF1–eRF3 translation termination complex that associates with UPF1 and SMG1-SMG8-SMG9 to form SURF and activate NMD	[53]
	*NCBP1*	CBP80	Component of the cap-binding complex (CBC) directly binds to UPF1, promoting the interaction with UPF2 to form SURF and activate NMD	[56,57,58]
	*NCBP2*	CBP20	Component of the CBC is essential for the stability of complex	[56,57,58]
	*EIF4E*	eIF4E	Eukaryotic translation initiation factor 4E binds to UPF1 and activates NMD	[59]
	*SMG1*	ATX	Suppressor of morphogenesis in genitalia-1 associate with SMG8-SMG9 to form the SMG1c kinase complex that catalyzes UPF1 phosphorylation	[53,60,61]
	*SMG8*		Suppressor of morphogenesis in genitalia-8 and suppressor of morphogenesis in genitalia-9 are co-factors that regulate SMG1 kinase activity	[53,60,61]
	*SMG9*			
	*DHX34*	KIAA0134	RNA helicase DHX34 binds SMG1 and promotes UPF1 phosphorylation, triggering the conversion from the SURF to the DECID complex	[62,63,64]
	*RUVBL1*	RVB1	AAA-ATPases RUVBL1 and RUVBL2 form a hetero-hexameric ring promoting the transition from SURF to the DECID complex	[65,66]
	*RUVBL2*	RVB2		
mRNA decay	*SMG5*	EST1B	Suppressor of morphogenesis in genitalia-5 and -7 form a heterodimer that binds p-UPF1 and recruit decapping enzymes, the CCR4-NOT complex (through CNOT8) and PP2A	[54,67,68]
	*SMG7*	EST1C		
	*SMG6*	EST1A	Endonuclease that interacts both with UPF1 and p-UPF1 and cleaves NMD targets close to the PTC	[54,69,70,71]
	*CNOT8*	POP2 CAF1	CCR4-NOT transcription complex subunit 8 is recruited by SMG7 to degrade NMD targets	[72]
	*DCP1A*		mRNA-decapping enzyme 1A is a decapping activator and together with PNRC2 stimulate the decapping activity of DCP2	[73]
	*PNRC2*		Proline rich nuclear receptor coactivator 2 binds p-UPF1 and stimulate the decapping activity of DCP2	[73]
	*DCP2*		Decapping protein engaged in the 5′→3′ mRNA degradation	[74]
	*MOV10*	gb110	RNA helicase contributes to degradation of UPF1-regulated transcripts	[75]
	*PPP2CA*	PP2AC	Protein phosphatase 2 (PP2A) promotes dephosphorylation of UPF1; both structural (*PPP2R1A*) and catalytic (*PPP2CA*) subunits of PP2A interact with SMG5	[54,67,68]
	*PPP2R1A*	PP2AA		
	*XRN1*		Exonuclease involved in 5′→3′ mRNA degradation	[74]
	*DIS3L*	DIS3L1	Core exosome-associated factor involved in the 3′→5′ mRNA degradation	[76]
	*DIS3L2*		Exoribonuclease that degrades mRNA from 3′→5′	[77]
	*EXOSC10*	PM/Scl100 Rrp6p	Exosome catalytic subunit involved in the 3′→5′ mRNA degradation	[74]
	*PARN*		Ribonuclease engaged in the 3′→5′ mRNA degradation	[74]
ER–NMD	*NBAS*	NAG	Protein involved in Golgi-to-endoplasmic reticulum (ER) retrograde transport recruits UPF1 to the membrane of the ER and regulates a subset of NMD targets translated at the ER	[62,78]
Regulator	*PABPC1*	PABP1	Polyadenylate-binding protein 1 inhibits the interaction of UPF1 with eRF3, repressing NMD	[79,80]
	*EIF4G1*	EIF4G	Eukaryotic initiation factor 4G inhibits NMD	[81,82]
	*UPF3A*	UPF3	Up-frameshift protein 3A compete with UPF3B for UPF2-binding and inhibits NMD	[83]
	*FMR1*	FMRP	Fragile X mental retardation protein binds directly to UPF1 and acts as an NMD repressor	[28]
	*EIF3E*	INT6 EIF3S6	Eukaryotic translation initiation factor 3 subunit E is a non-core eIF3 subunit that interacts with UPF2 and triggers NMD	[84]
	*SRSF1*	SFRS1	Serine/arginine-rich splicing factor 1 promotes NMD by enhancing UPF1-binding to the mRNA in the nucleus and it is also involved in UPF1 dephosphorylation	[85]
	*SEC13*		GNL2 and SEC13 are conserved NMD factors that regulate endogenous NMD targets but their exact role is unknown	[86]
	*GNL2*	Ngp-1		

^(1)^ Gene annotation according to HGNC. ^(2)^ Previous SYMBOL or alias reported by HGNC database or identifier found in literature review. Legend: NMD, Nonsense-Mediated mRNA Decay; PTC, Premature Termination Codon; EJC, Exon Junction Complex; SURF, SMG-1–Upf1–eRF1–eRF3 complex; DECID, Decay Inducing Complex; ER-NMD, NMD response at the Endoplasmic Reticulum; p-UPF1, phosphorylated UPF1.

**Table 2:** NMD genes disrupted by CNVs identified in a population of 3570 ASD patients.

NMD Group	Gene	Location	CNV Type	Gene Region	Protein Domains Affected	ASD N ^(1)^
EJC	*RBM8A*	1q21.1	Deletion	Whole gene	All domains	1
	*RBM8A*	1q21.1	Duplication	Whole gene	All domains	3
SURF-DECID	*UPF2*	10p14	Duplication	Partial	MIF4G domains	1
	*UPF3B*	Xq24	Duplication	Whole gene	All domains	1
	*GSPT1*	16p13.13	Duplication	Whole gene	All domains	1
	*NCBP2*	3q29	Deletion	Whole gene	All domains	1
	*DHX34*	19q13.32	Deletion	Whole gene	All domains	1
	*RUVBL2*	19q13.33	Duplication	Partial	DI domain	1
mRNA decay	*DIS3L*	15q22.31	Duplication	Whole gene	All domains	1
	*DIS3L2*	2q37.1	Deletion	Partial	Part of CSD2 and RNB domains	1
	*DIS3L2*	2q37.1	Duplication	Partial	RNB and C-terminal S1 domain	1
	*EXOSC10*	1p36.22	Deletion	Partial	PMC2NT, EXO1 and HRCD domains	1
	*EXOSC10*	1p36.22	Duplication	Partial	EXO and HRCD domain	1
	*MOV10*	1p13.2	Duplication	Whole gene	All domains	2
	*PARN*	16p13.12	Deletion	Partial	All domains	1
	*PPP2R1A*	19q13.41	Duplication	Partial	PP2A subunit B binding	1
	*SMG6*	17p13.3	Duplication	Partial	PIN domain	1
	*XRN1*	3q23	Duplication	Partial	XRN1 SH3-like domain	13
ER-NMD	*NBAS*	2p24.3	Duplication	Partial	Sec39-like domain	1
Regulator	*FMR1*	Xq27.3	Deletion	Partial	KH2, NES and RGG domains	1
	*UPF3A*	13q34	Duplication	Whole gene	All domains	2
	*UPF3A*	13q34	Duplication	Partial	EJC-binding domain	1

^(1)^ ASD N—number of ASD patients carrying CNVs.

**Table 3:** Numbers of SNVs or CNVs identified within protein domains important for NMD in 258 ASD patients. A total of 136 genetic variants in 23 NMD genes were identified.

NMD Group	Gene	Location	SNVs ^(1)^		CNVs ^(1)^	N ASD ^(2)^	pLI	mis_Z	SFARI ^(3)^
			LoF	MISPD					
EJC	*EIF4A3*	17q25.3		1		1	1.00	4.02	
	*RBM8A*	1q21.1			2	4	0.57	2.16	1q21.1 region
	*CASC3*	17q21.1		3		3	0.61	1.32	
	*ICE1*	5p15.32	2	7		10	1.00	0.73	
SURF-DECID	*UPF1*	19p13.11		4		4	1.00	5.63	
	*UPF2*	10p14	1	3	1	11	1.00	3.19	category 3
	*UPF3B*	Xq24	1	3	1	7	0.98	1.84	category 1
	*ETF1*	5q31.2	1	3		6	1.00	4.39	
	*GSPT1*	16p13.13		4	1	7	1.00	3.32	
	*DHX34*	19q13.32	2	12	1	25	0.00	−0.08	
	*RUVBL1*	3q21.3		1		1	1.00	3.39	
	*RUVBL2*	19q13.33		1	1	2	1.00	3.11	
	*SMG1*	16p12.3	1	12		13	1.00	3.30	
	*SMG8*	17q22		6		8	0.01	1.73	
	*SMG9*	19q13.31	1	1		4	0.00	1.60	
mRNA decay	*SMG5*	1q22		2		2	0.01	1.13	
	*SMG6*	17p13.3		2	1	6	0.98	0.18	category 3
	*SMG7*	1q25.3		8		14	1.00	2.19	
ER-NMD	*NBAS* ^(4)^	2p24.3	5	20	1	99	0.00	−0.87	
Regulator	*UPF3A*	13q34	1	2	2	11	0.00	−0.60	
	*SRSF1*	17q22		1		1	0.98	3.96	
	*SEC13* ^(4)^	3p25.3		2		2	0.02	0.62	
	*GNL2* ^(4)^	1p34.3	1	11		17	0.00	0.28	

^(1)^ Numbers of SNVs and CNVs located within regions necessary for NMD function. ^(2)^ Numbers of ASD patients carrying SNVs and CNVs. ^(3)^ Genes and CNVs associated with ASD, described in SFARI Gene database (https://gene.sfari.org/ accessed on 8 March 2021). ^(4)^ These genes do not have the described NMD domains. Legend: SNVs, Single Nucleotide Variants; CNVs, Copy Number Variants; pLI, probability of being loss-of-function intolerant; mis_Z, missense Z scores.

## Data Availability

Data sharing not applicable.

## Supplementary Materials

The following are available online at https://www.mdpi.com/article/10.3390/biomedicines10030665/s1, Table S1: List of Genes obtained by querying “nonsense mediated decay” in AmiGO database; Table S2: List of proteins involved in NMD pathway (R-HSA-927802) annotated by Reactome; Table S3: Rare Variants identified in NMD genes in ASD patients; Table S4: Rare, high-confidence CNVs targeting NMD genes in ASD samples; Table S5: List of ASD risk genes from Sfari database that are experimentally validated NMD targets.
